# Factors Associated With Health Care Professionals’ Choice to Practice in Rural Minnesota

**DOI:** 10.1001/jamanetworkopen.2023.10332

**Published:** 2023-05-04

**Authors:** Teri Fritsma, Carrie Henning-Smith, Jacqueline L. Gauer, Faizel Khan, Mark E. Rosenberg, Kirby Clark, Elizabeth Sopdie, Angela Sechler, Michael A. Sundberg, Andrew P. J. Olson

**Affiliations:** 1 Minnesota Department of Health, St Paul; 2 University of Minnesota Rural Health Research Center, University of Minnesota School of Public Health, Minneapolis; 3 Medical Education Outcomes Center, Office of Medical Education, University of Minnesota Medical School, Minneapolis; 4 Department of Family Medicine and Community Health, University of Minnesota Medical School, Minneapolis; 5 Rural and Metropolitan Physician Associate Programs, Office of Medical Education, University of Minnesota Medical School, Minneapolis; 6 Division of Hospital Medicine, Department of Medicine, University of Minnesota Medical School, Minneapolis; 7 Division of Pediatric Hospital Medicine, Department of Pediatrics, University of Minnesota Medical School, Minneapolis

## Abstract

**Importance:**

Rural health inequities are due in part to a shortage of health care professionals in these areas.

**Objective:**

To determine the factors associated with health care professionals’ decisions about where to practice.

**Design, Setting, and Participants:**

This prospective, cross-sectional survey study of health care professionals in Minnesota was administered by the Minnesota Department of Health from October 18, 2021, to July 25, 2022. Advanced practice registered nurses (APRNs), physicians, physician assistants (PAs), and registered nurses (RNs) renewing their professional licenses were eligible.

**Exposures:**

Individuals’ ratings on survey items related to their choice of practice location.

**Main Outcomes and Measures:**

Rural or urban practice location as defined by the US Department of Agriculture’s Rural-Urban Commuting Area typology.

**Results:**

A total of 32 086 respondents were included in the analysis (mean [SD] age, 44.4 [12.2] years; 22 728 identified as female [70.8%]). Response rates were 60.2% for APRNs (n = 2174), 97.7% for PAs (n = 2210), 95.1% for physicians (n = 11 019), and 61.6% for RNs (n = 16 663). The mean (SD) age of APRNs was 45.0 (10.3) years (1833 [84.3%] female); PAs, 39.0 (9.4) years (1648 [74.6%] female); physicians, 48.0 (11.9) years (4455 [40.4%] female); and RNs, 42.6 (12.3) years (14 792 [88.8%] female). Most respondents worked in urban (29 456 [91.8%]) vs rural (2630 [8.2%]) areas. Bivariate analysis suggested that family considerations are the most important determinant of practice location. Multivariate analysis revealed that having grown up in a rural area was the strongest factor associated with rural practice (odds ratio [OR] for APRNs, 3.44 [95% CI, 2.68-4.42]; OR for PAs, 3.75 [95% CI, 2.81-5.00]; OR for physicians, 2.44 [95% CI, 2.18-2.73]; OR for RNs, 3.77 [95% CI, 3.44-4.15]). When controlling for rural background, other associated factors included the availability of loan forgiveness (OR for APRNs, 1.42 [95% CI, 1.19-1.69]; OR for PAs, 1.60 [95% CI, 1.31-1.94]; OR for physicians, 1.54 [95% CI, 1.38-1.71]; OR for RNs, 1.20 [95% CI, 1.12-1.28]) and an educational program that prepared for rural practice (OR for APRNs, 1.44 [95% CI, 1.18-1.76]; OR for PAs. 1.70 [95% CI, 1.34-2.15]; OR for physicians, 1.31 [95% CI, 1.17-1.47]; OR for RNs, 1.23 [95% CI, 1.15-1.31]). Autonomy in one’s work (OR for APRNs, 1.42 [95% CI, 1.08-1.86]; OR for PAs, 1.18 [95% CI, 0.89-1.58]; OR for physicians, 1.53 [95% CI, 1.31-1.78]; OR for RNs, 1.16 [95% CI, 1.07-1.25]) and a broad scope of practice (OR for APRNs, 1.46 [95% CI, 1.15-1.86]; OR for PAs, 0.96 [95% CI, 0.74-1.24]; OR for physicians, 1.62 [95% CI, 1.40-1.87]; OR for RNs, 0.96 [95% CI, 0.89-1.03]) were important factors associated with rural practice. Lifestyle and area considerations were not associated with rural practice; family considerations were associated with rural practice for RNs only (OR for APRNs, 0.97 [95% CI, 0.90-1.06]; OR for PAs, 0.95 [95% CI, 0.87-1.04]; OR for physicians, 0.92 [95% CI, 0.88-0.96]; OR for RNs, 1.05 [95% CI, 1.02-1.07]).

**Conclusions and Relevance:**

Understanding the interconnected factors involved in rural practice requires modeling relevant factors. The findings of this survey study suggest that loan forgiveness, rural training, autonomy, and a broad scope of practice are factors associated with rural practice for most health care professionals. Other factors associated with rural practice vary by profession, suggesting that there may not be a one-size-fits-all approach to recruitment of rural health care professionals.

## Key Points

**Question:**

What are the factors associated with health care workers’ decisions regarding where they live and work?

**Findings:**

This cross-sectional survey study of 32 086 health care professionals from Minnesota showed that most work in urban areas. Commonly cited factors associated with working in rural areas included growing up in a rural area, participation in a rural training program, practice characteristics and autonomy, and the availability of loan repayment programs, with the relative importance of these factors varying between professions.

**Meaning:**

These findings suggest that to mitigate health care worker shortages, attention must be given to the factors associated with the decision to live and work in rural areas, and profession-specific recruitment approaches are likely to be most effective.

## Teaser

This cross-sectional survey study identifies factors associated with health care professionals choosing to practice in rural areas and how these factors vary across different profession types.

## Introduction

Rural inequities in health are well documented and have worsened during the COVID-19 pandemic.^1,2,3,4^ One contributing factor to poorer health outcomes among rural residents vs urban residents is access to health care.^2,3,5^ Rural residents face several barriers in accessing care, including transportation challenges, longer distances, facility closures, higher rates of residents without insurance, and more limited access to broadband internet and other telehealth capabilities.^2,5,6,7,8^ Another challenge is health care workforce availability in rural areas. According to the US Bureau of Health Workforce, as of November 2, 2022, 5357 primary care health professional shortage areas were located in rural areas, constituting 66% of all shortage areas, with an additional 403 (5%) located in partially rural areas.^9^ Moreover, in Minnesota, approximately 15% of the population lives in rural areas, while only about 8% of the health care workforce practices in those same areas.^10^

There is a growing but still limited body of evidence regarding the factors that influence recruitment and retention of rural health care professionals. Some global evidence suggests that regulatory measures, as well as attracting health care professionals who grew up in or trained in rural areas, positively impact rural recruitment.^11^ In the US, a 2015 systematic review found that growing up in a rural community was the most consistent factor associated with choice of a rural practice location and that training efforts seemed effective at increasing the likelihood of future rural practice.^12^

Medical education institutions have explored a variety of strategies to increase the rural physician workforce across the educational spectrum, from admissions policies that prioritize students from rural backgrounds to offering rural training during medical school and residency.^13^ Observational data suggest that medical students who participate in a rural training program are more likely to practice in rural primary care compared with their counterparts without these experiences.^14^

Other factors such as lifestyle, family considerations, financial incentives, practice characteristics, availability of loan forgiveness, and personal characteristics also influence the decision to practice in rural areas.^15^ These factors are multifaceted, nuanced, and interrelated, and studies investigating the relative influence of these various factors on rural practice are lacking. Further clarity is needed to understand which factors leading to rural practice are modifiable and which are fixed. This information is needed to inform policy makers, health systems, educational institutions, and other stakeholders in the allocation of resources to mitigate rural workforce shortages. This study aims to identify which factors are associated with influencing a health care professional to choose to practice in a rural area, as well as how these factors vary across different profession types.

## Methods

This survey study was approved by the institutional review boards of both the University of Minnesota and the Minnesota Department of Health (MDH). Respondents were informed prior to the survey that their responses would be kept private, would not influence license renewal, and would not be shared in anything but aggregate form. The study relied on a novel data set that merges 2 distinct record sources: (1) administrative data from Minnesota’s Nursing and Medical Practice licensing boards and (2) data from the MDH Healthcare Workforce Survey of active health care professionals, conducted from October 18, 2021, through July 25, 2022. This study followed the American Association for Public Opinion Research (AAPOR) reporting guideline.

### Administrative Data From Licensing Boards

The MDH receives a monthly transfer of administrative records from the Board of Nursing, which governs the licensing of nurses, and the Board of Medical Practice, which governs the licensing physicians and physician assistants (PAs) in Minnesota. The study population included all licensed physicians, PAs, advanced practice registered nurses (APRNs), and registered nurses (RNs) who had active Minnesota licenses as of July 25, 2022. At that time, a total of 26 874 physicians, 3922 PAs, 10 946 APRNs, and 111 376 RNs had active Minnesota licenses. The relevant variables from the licensing boards’ records included current practice address, age, and date of birth, described in more detail below.

### Data From the MDH License Renewal Survey

As mandated in Minnesota statute, the MDH surveys all physicians, PAs, APRNs, and RNs at the time of license renewal. Physicians and PAs renew licenses annually and nurses renew them biennially online. All health care professionals are required to renew their licenses during their birth month, making the timing of the renewal and survey responses random with respect to all factors except birth month. First-time license applicants are not surveyed. Physicians and PAs must complete the survey before being able to pay the license renewal fee, whereas nurses take the survey after they have paid the fee, historically causing a slightly lower response rate among nurses. Effectively, this method means that during the course of 1 year for physicians and PAs and 2 years for nurses, survey responses are collected from most licensees in Minnesota. The data for this study were collected from October 18, 2021, through July 25, 2022; data were analyzed at interim points throughout the study period, and the sample size was verified to be large enough to have power to make inferences without significant change from adding more responses. Board licensing data and MDH survey data were merged and linked using the licensee’s license number, a unique identifier across the 2 data sets.

### Variables and Measurement

The primary outcome, or dependent variable, in this study was whether or not the respondent was practicing in a rural area. We operationalized this using the US Department of Agriculture’s Rural-Urban Commuting Area (RUCA) typology, which incorporates measures of population density, urbanization, and daily commuting patterns to categorize the level of rurality for regions at the level of US census tracts.^16^ In this study, the address that each licensee reported to their licensing board was the basis for designating their RUCA. When reporting an address, respondents must indicate the address type (practice, mailing, home, or other). Working physicians and PAs are required to report their primary practice address, whereas nurses are permitted to report a mailing address, most of which are their employment location, and the remainder (a small but unknowable share) are private residences. When the address is a private residence, this analysis relies on the assumption that the respondent’s residence and location of practice are in the same RUCA.

We cleaned and refined these address data by correcting misspellings, typos, and street directionals; replacing PO boxes with physical addresses where possible; and correcting erroneous city names and zip codes. After cleaning, we geocoded each address to the census tract level and assigned each census tract to a RUCA using the geographic information software ArcMap, version 10.8 (Esri). The 4 resulting RUCA categories are urban, micropolitan or large town, small town or small rural, and isolated rural. We then collapsed these categories to 2: urban (which includes the RUCA urban and micropolitan or large town categories) and rural (which includes the RUCA small town or small rural and isolated rural categories). Respondents who reported out-of-state addresses were excluded from the analysis because they could not be geocoded in Minnesota.

Prior literature shows that having grown up in a rural area makes a person far more likely to choose a rural area in which to live and practice, making the region in which a person grew up the key control variable in this study.^11^ The MDH survey measures this with the question, “Which of these best describes the area in which you grew up?” Response options were “large metropolitan or surrounding,” “small city,” and “small town or rural area.” This classification differs from the 4-category RUCA classification. We opted to operationalize the concept this way for several reasons. First, we relied on respondents’ self-report to determine the type of area in which they grew up. Through our respondent interviews (described below), it became apparent that respondents did not easily distinguish between “small town” and “isolated rural” consistently in the same way that the RUCA classification does, and we concluded that an ordinal variable with 3 categories would likely yield more reliable measurement than a 4-category variable that tried to mimic the RUCA categories. Respondents’ age was another important control variable in this study because rural health care professionals are older, on average, than urban health care professionals. Age is also a proxy for years in practice, which could also be related to how a health care professional makes decisions on where to practice. We coded age using the respondent’s birth date as reported to the licensing board.

The key set of independent variables in this research were the considerations that respondents weighed when deciding on the type of area (rural vs urban) in which they work. To measure these considerations, we began by identifying a list of items already identified in both the literature and policy realms as being related to the choice to practice in a rural area. This list included 5 general categories: family considerations, practice considerations, financial and loan forgiveness incentives, education and training experiences, and characteristics of the area itself. We then wrote a draft set of survey questions to capture various dimensions of each of these 5 broad considerations.

We conducted 22 preliminary interviews with various health care professionals to identify inevitable validity problems with our draft questions. Those interviews guided us to revise questions where the intent behind a given question was not translating correctly to respondents or where there was redundancy among our questions.

Based on this work, our final survey instrument included 16 unique items that respondents were asked to rate. The survey asked, “Think back to how you made the decision to live in your general area. How important were each of the following considerations?” with response options including “very important,” “somewhat important,” “not important at all,” and “did not apply to me.” The individual items included measures across the 5 general categories we identified at the outset (family considerations, practice considerations, financial and loan forgiveness incentives, education and training experiences, and characteristics of the area itself). The full final set of individual items are shown in Supplement 1.

Based on intercorrelations among the individual responses, we created 2 scaled variables. A combined family considerations variable included responses to 2 of the individual survey items: “whether my partner/spouse would have job opportunities” and “whether this would be a good place to raise children.” The combined lifestyle area considerations variable included responses to 3 of the individual survey items: “whether the community here would be a good fit for me,” “the quality of life in this area,” and “the lifestyle in this area.” The family considerations variable ranges from 1 to 9 and the area considerations variable ranges from 1 to 10, with higher scores indicating greater importance. Combining these individual items greatly simplifies the interpretation of our findings.

The survey asked a final summary question, in part to confirm and validate the preceding set of responses: “When thinking about the area in which you live (major metro area; small city; small town/rural), which of these would you say influenced your decision the most?” Here, response options included: “family considerations,” “the way I could practice/work in this area,” “education/training experiences,” “things about the area itself,” “financial incentives,” and “something else.”

### Statistical Analysis

We generated descriptive statistics to show characteristics of our sample and the distributions on the independent and dependent variables by the 4 profession types. We then performed a univariate analysis showing rural respondents’ rating of the most important consideration based on the survey’s final summary question (described above). Last, we developed multivariate logistic regression models, with practice location as the dependent variable for each profession, for all respondents (regardless of whether they currently worked in an urban or rural practice). All analyses were conducted in SPSS, version 29.0.0 (IBM Corporation) with 1-sided hypothesis tests; *P* < .05 indicated statistical significance.

## Results

A total of 32 086 respondents were included in the analysis (mean [SD] age, 44.4 [12.2] years; 22 728 identified as female [70.8%] and 9358 [29.2%] as male). With regard to race and ethnicity, 1347 respondents (4.2%) were African or African American; 89 (0.3%), American Indian or Alaska Native; 2426 (8.6%), Asian; 464 (1.4%), Hispanic or Latino; 26 109 (81.4%), White; 985 (3.1%), more than 1 race or ethnicity; and 666 (2.1%), other race or ethnicity. These data helped to describe the population of Minnesota health care professionals but were not used as a covariate. Table 1 provides basic descriptive data. The professions differed slightly in terms of age, sex, and race and ethnicity. Survey response rates were 60.2% for APRNs (n = 2714), 95.1% for physicians (n = 11 019), 97.7% for PAs (n = 2210), and 61.6% for RNs (n = 16 663). With these response rates, the results in this study represent 32% of all Minnesota APRNs, 63% of all Minnesota physicians, 67% of all Minnesota PAs, and 28% of all Minnesota RNs. In our sample, 560 APRNs (19.7%), 3666 physicians (23.8%), 278 PAs (11.0%), and 4274 RNs (17.8%) reported out-of-state addresses and were excluded from the analysis. A total of 1833 APRNs (84.3%), 1648 PAs (74.6%), and 14 792 RNs (88.8%) identified as female. Most physicians identified as male (6564 [59.5%]) and were the most racially and ethnically diverse group of health care professionals. Profession groups differed on the distribution of where they grew up, with physicians most likely to report growing up in urban areas and RNs most likely to report growing up in rural areas. As shown in Table 1, health care professionals were distributed unequally between urban and rural areas of the state, with fewer than 10% of health care professionals in rural practice (2630 [8.2%]). Table 2 shows the univariate distribution for key control and independent variables. As shown, health care professionals rated many factors as very important in influencing their decision about where to practice regardless of whether they worked in an urban or a rural area. Family considerations were rated relatively similarly across all professions (mean [SD] score of 9 possible: 7.5 [2.3] for APRNs and PAs, 7.0 [2.5] for physicians, and 7.3 [2.4] for RNs). Lifestyle and area considerations were also rated similarly across all professions (mean [SD] score of 10 possible: 8.5 [2.1] for APRNs, 8.5 [1.9] for PAs, 8.3 [2.2] for physicians, and 8.4 [2.1] for RNs). With respect to other considerations, autonomy in work (1106 ARRNs [51.5%], 974 PAs [44.8%], 5304 physicians [48.7%], and 6559 RNs [39.8%]) and having a broad scope of practice (736 APRNs [34.1%], 612 PAs [28.1%], 3937 physicians [36.1%], and 4276 RNs [25.9%]) were very important when determining where to work. Internship, clinical training, or residency was rated as very important by 611 ARPNs (28.3%), 436 PAs (20.0%), 3659 physicians (33.6%), and 3574 RNs (21.7%).

**Table 1.** Demographic Characteristics

Characteristic	Health care profession^a^			
	APRNs (n = 2174)	PAs (n = 2210)	Physicians (n = 11 019)	RNs (n = 16 663)
Age, mean (SD), y	45.0 (10.3)	39.0 (9.4)	48.0 (11.9)	42.6 (12.3)
Sex				
Male	341 (15.7)	551^b^ (24.9)	6564 (59.5)	1871 (11.2)
Female	1833 (84.3)	1648 (74.6)	4455 (40.4)	14 792 (88.8)
Race and ethnicity				
African or African American	95 (4.4)	28 (1.3)	335 (3.0)	889 (5.3)
American Indian or Alaska Native	7 (0.3)	2 (0.1)	26 (0.2)	54 (0.3)
Asian	53 (2.4)	65 (2.9)	1684 (15.3)	624 (3.7)
Hispanic or Latino	17 (0.8)	14 (0.6)	224 (2.0)	209 (1.3)
White	1917 (88.2)	2023 (91.5)	8009 (72.6)	14 160 (85.0)
Multiple races	44 (2.0)	46 (2.1)	421 (3.8)	474 (2.8)
Other race or skipped question	41 (1.9)	32 (1.4)	340 (3.1)	253 (1.5)
Practice location				
Rural	192 (8.8)	162 (7.3)	551 (5.0)	1725 (10.4)
Urban	1982 (91.2)	2048 (92.7)	10 488 (95.0)	14 938 (89.6)

Abbreviations: APRNs, advanced practice registered nurses; PAs, physician assistants; RNs, registered nurses.

^a^ Unless otherwise indicated, data are expressed as No. (%) of professionals. Percentages have been rounded and may not total 100.

^b^ Data were missing for 1 PA.

**Table 2.** Distribution on Independent Variables

Variable	Health care profession^a^			
	APRNs (n = 2174)	PAs (n = 2210)	Physicians (n = 11 019)	RNs (n = 16 663)
Where did you grow up?				
Large metropolitan or surrounding	793 (36.6)	874 (39.7)	5355 (48.9)	5883 (35.5)
Small city	509 (23.5)	567 (25.8)	2753 (25.1)	3438 (20.7)
Small town or rural area	863 (39.9)	758 (34.5)	2851 (26.0)	7271 (43.8)
Scaled variables, mean (SD)				
Family considerations^b^	7.5 (2.3)	7.5 (2.3)	7.0 (2.5)	7.3 (2.4)
Lifestyle and area considerations^c^	8.5 (2.1)	8.5 (1.9)	8.3 (2.2)	8.4 (2.1)
**Ordinal variables**				
Having autonomy in my work				
Very important	1106 (51.5)	974 (44.8)	5304 (48.7)	6559 (39.8)
Somewhat important	790 (36.8)	939 (43.2)	4256 (39.1)	6923 (42.0)
Not important	141 (6.6)	164 (7.5)	608 (5.6)	1644 (10.0)
Did not apply to me	110 (5.1)	99 (4.5)	723 (6.6)	1359 (8.2)
Having a broad scope of practice				
Very important	736 (34.1)	612 (28.1)	3937 (36.1)	4276 (25.9)
Somewhat important	981 (45.5)	1093 (50.1)	4926 (45.2)	7590 (45.9)
Not important	223 (10.3)	260 (11.9)	1043 (9.6)	2292 (13.9)
Did not apply to me	218 (10.1)	215 (9.9)	989 (9.1)	2373 (14.4)
Whether I could (or did) receive loan forgiveness				
Very important	313 (14.5)	275 (12.6)	946 (8.7)	2110 (12.8)
Somewhat important	411 (19.0)	415 (19.0)	1703 (15.6)	2962 (17.9)
Not important	481 (22.3)	596 (27.3)	3270 (30.0)	3608 (21.8)
Did not apply to me	954 (44.2)	901 (41.2)	4985 (45.7)	7838 (47.5)
An internship, clinical training, or residency prepared me				
Very important	611 (28.3)	436 (20.0)	3659 (33.6)	3574 (21.7)
Somewhat important	679 (31.5)	689 (31.6)	3489 (32.0)	5160 (31.3)
Not important	287 (13.3)	375 (17.2)	1630 (15.0)	2449 (14.8)
Did not apply to me	579 (26.9)	677 (31.1)	2121 (19.5)	5318 (32.2)
My educational program prepared me				
Very important	388 (18.0)	261 (12.0)	1657 (15.2)	2686 (16.3)
Somewhat important	629 (29.2)	685 (31.4)	3407 (31.3)	4525 (27.4)
Not important	512 (23.7)	609 (27.9)	2899 (26.6)	3668 (22.2)
Did not apply to me	628 (29.1)	627 (28.7)	2934 (26.9)	5640 (34.1)
A financial incentive, such as higher pay or a hiring bonus				
Very important	560 (25.9)	397 (18.2)	1783 (16.3)	4166 (25.2)
Somewhat important	790 (36.6)	845 (38.8)	4056 (37.2)	5640 (34.1)
Not important	376 (17.4)	479 (22.0)	3171 (29.1)	2643 (16.0)
Did not apply to me	433 (20.1)	458 (20.1)	1901 (17.4)	4086 (24.7)
Whether I could specialize in certain types of care in this area				
Very important	752 (34.9)	712 (32.7)	4378 (40.2)	4842 (29.3)
Somewhat important	821 (38.1)	876 (40.3)	3622 (33.2)	6539 (39.6)
Not important	353 (16.4)	367 (16.9)	1559 (14.3)	2861 (17.3)
Did not apply to me	229 (10.6)	221 (10.2)	1339 (12.3)	2267 (13.7)
Working with certain types of patients				
Very important	669 (31.0)	460 (21.1)	2812 (25.8)	4378 (26.5)
Somewhat important	901 (41.7)	1008 (46.3)	4773 (43.9)	6566 (39.8)
Not important	408 (18.9)	553 (25.4)	2390 (22.0)	3623 (22.0)
Did not apply to me	181 (8.4)	158 (7.3)	909 (8.4)	1937 (11.7)

Abbreviations: APRNs, advanced practice registered nurses; PAs, physician assistants; RNs, registered nurses.

^a^ Unless otherwise indicated, data are expressed as No. (%) of professionals responding to the survey item. Percentages have been rounded and may not total 100.

^b^ Scores range from 1 to 9, with higher scores indicating more importance placed on this consideration.

^c^ Scores range from 1 to 10, with higher scores indicating more importance placed on this consideration.

**Figure 1.**
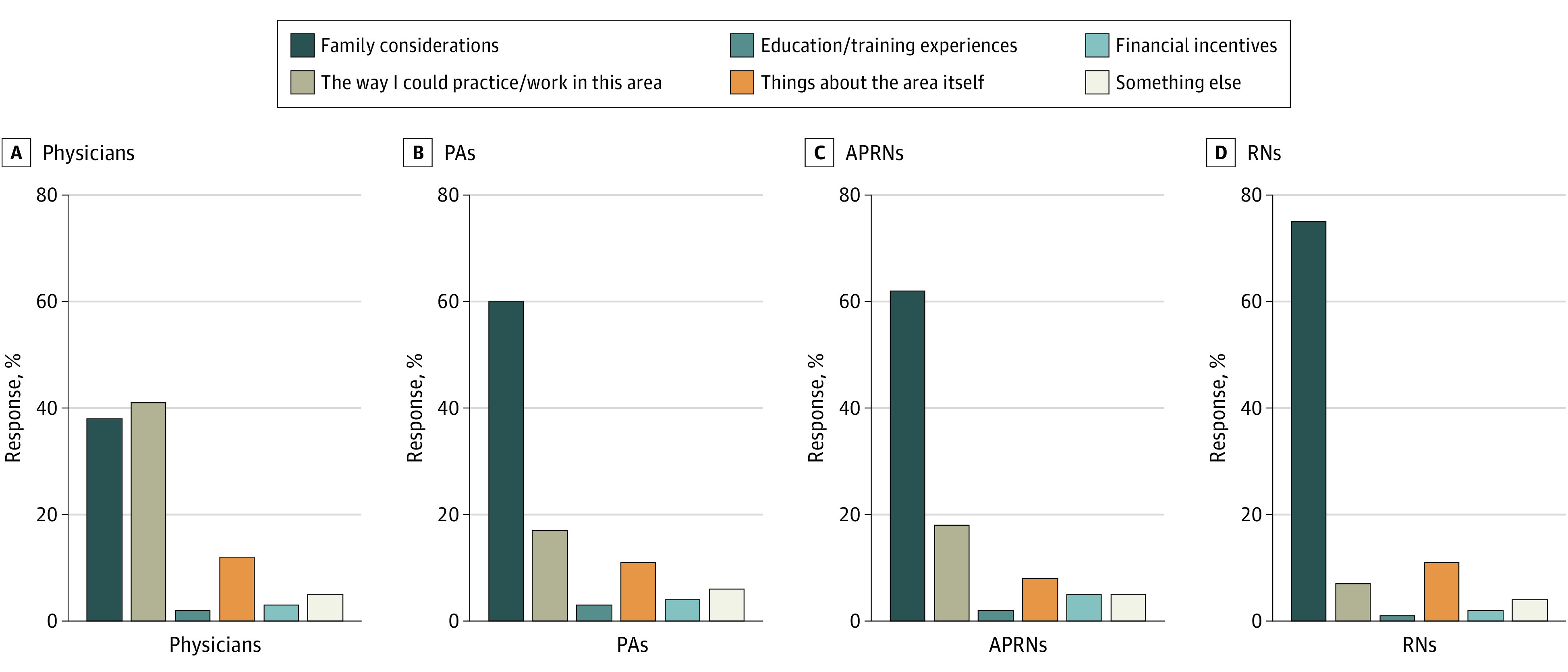
Most Important Consideration in Deciding Where to Live and Work Among Rural Health Care Professionals in Minnesota, 2022 APRNs indicates advanced practice registered nurses; PAs, physician assistants; and RNs, registered nurses. Group differences are statistically significant (*P* < .01) based on the χ^2^ test used to reject the null hypothesis that all the groups have the same distribution.

Figure 1 shows univariate analysis on only rural respondents’ answers to the aforementioned summary question—the single most important factor respondents considered when choosing where to live. Rural physicians were the only group to choose practice characteristics (223 [40.5%]), while APRNs (118 [61.5%]), PAs (96 [59.3%]), and RNs (1286 [74.6%]) most frequently chose family considerations. Of note, financial considerations were selected as most important by 5% or less of each group.

**Figure 2.**
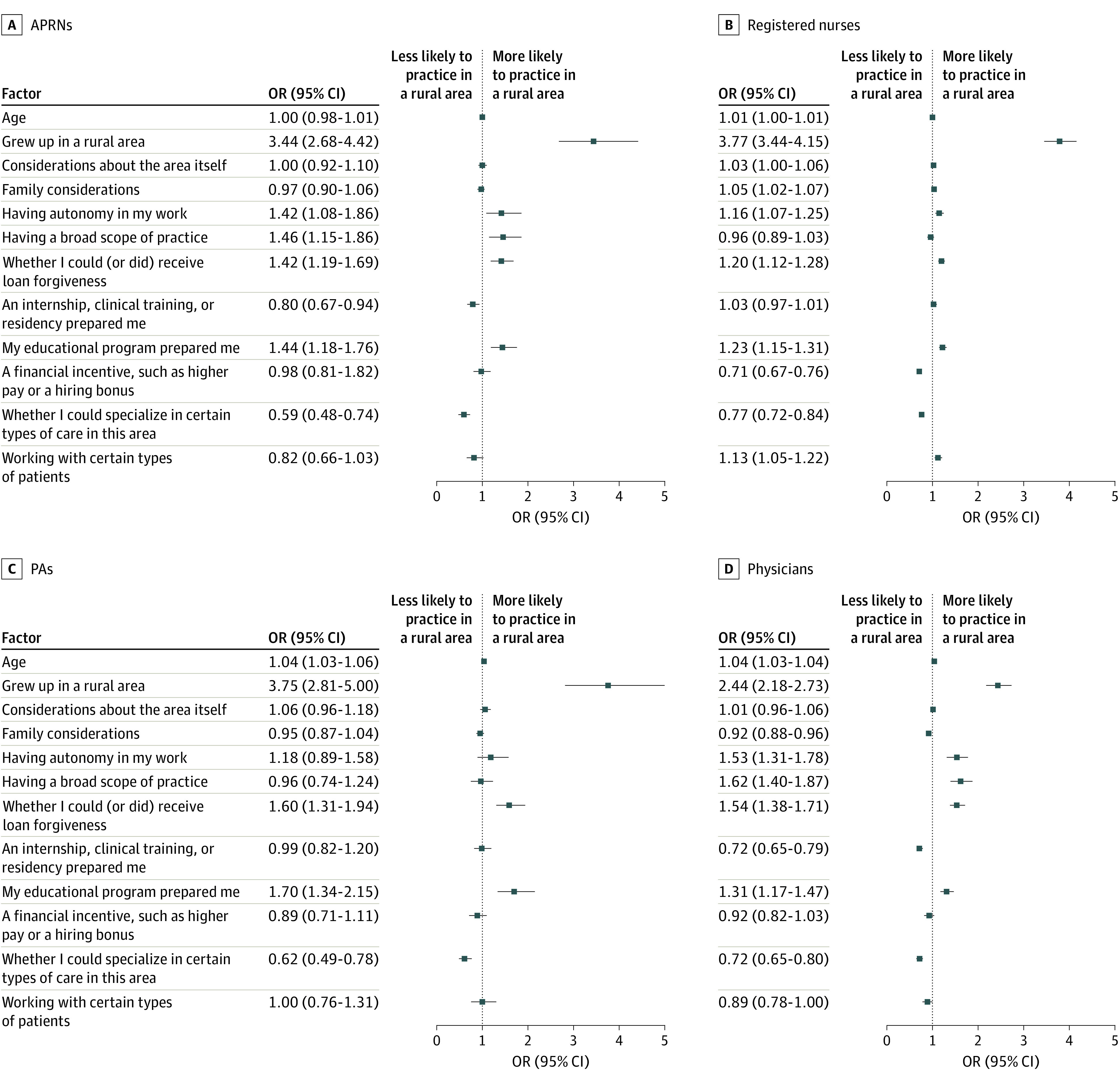
Multivariate Analysis of the Factors Associated With the Choice of a Rural Practice Location for 4 Health Care Professions in Minnesota, 2022 APRNs indicates advanced practice registered nurses; OR, odds ratio; PAs, physician assistants.

Figure 2 presents the results from the more comprehensive multivariate logistic regression models that include all relevant factors—where health care professionals grew up as well as the various factors they considered when deciding where to practice. Each factor’s association with the odds of practicing in a rural area is shown. The most important factor across all professions associated with determining rural practice is growing up in a rural area (odds ratio [OR] for APRNs, 3.44 [95% CI, 2.68-4.42]; OR for PAs, 3.75 [95% CI, 2.81-5.00]; OR for physicians, 2.44 [95% CI, 2.18-2.73]; OR for RNs, 3.77 [95% CI, 3.44-4.15]). However, other associated factors across all profession groups included the availability of loan forgiveness (OR for APRNs, 1.42 [95% CI, 1.19-1.69]; OR for PAs, 1.60 [95% CI, 1.31-1.94]; OR for physicians, 1.54 [95% CI, 1.38-1.71]; OR for RNs, 1.20 [95% CI, 1.12-1.28]) and an educational program that prepared professionals for rural practice (OR for APRNs, 1.44 [95% CI, 1.18-1.76]; OR for PAs, 1.70 [95% CI, 1.34-2.15]; OR for physicians, 1.31 [95% CI, 1.17-1.47]; OR for RNs, 1.23 [95% CI, 1.15-1.31]). Having autonomy in work (OR for APRNs, 1.42 [95% CI, 1.08-1.86]; OR for PAs, 1.18 [95% CI, 0.89-1.58]; OR for physicians 1.53 [95% CI, 1.31-1.78]; OR for RNs, 1.16 [95% CI, 1.07-1.25]) and a broad scope of practice (OR for APRNs, 1.46 [95% CI, 1.15-1.86]; OR for PAs, 0.96 [95% CI, 0.74-1.24]; OR for physicians, 1.62 [95% CI, 1.40-1.87]; OR for RNs, 0.96 [95% CI, 0.89-1.03]) varied in relative importance across the 4 profession groups. Importantly, in the multivariate analysis, family considerations were associated with rural practice only for RNs (OR for APRNs, 0.97 [95% CI, 0.90-1.06]; OR for PAs, 0.95 [95% CI, 0.87-1.04]; OR for physicians, 0.92 [95% CI, 0.88-0.96]; OR for RNs, 1.05 [95% CI, 1.02-1.07]). This plainly reveals the potential limitations of a more simplified analysis that fails to control on relevant factors.

## Discussion

To effectively address rural health inequities—which stem, in part, from rural workforce shortages—it is critical to understand the factors that lead health care professionals to choose rural practice. In this cross-sectional survey study, we identified and measured a wide variety of those factors and then estimated the relative importance of each in predicting rural practice across 4 health care professions (APRNs, physicians, PAs, and RNs). The findings encompass responses from a large and representative sample of professionals in each of these fields in Minnesota, representing the most comprehensive study to date on this topic, to our knowledge. The work offers multiple important implications for future research, policy, educational programming, and other interventions aimed at decreasing rural health inequities.

Certain factors are common to all 4 professions that are associated with the choice of rural practice. Echoing other studies and the conventional wisdom, our findings indicate that growing up in a rural area is by far the most important determinant of having a current rural practice. While this is not surprising, it lends strong support to programs that engage rural residents in “grow-your-own” pipeline programs to enter health profession training programs.

Health care professionals in all 4 fields also indicate that educational programs with a rural focus influenced their decision to practice in a rural area, and the association between educational exposure and rural practice remains even after controlling for rural background. This finding has important implications for both the content and the location of educational programs. Butler et al^14^ described the positive effect of a rural undergraduate medical education program on rural practice. Our present findings suggest that programs focused specifically on equipping individuals to practice in a rural setting are effective at influencing individuals to practice in a rural area.

In addition, access to loan forgiveness is associated with choosing rural practice over and above having a rural background, and this is true across all 4 profession groups. This suggests that rural health care systems as well as federal, tribal, and state governments could influence individuals to practice in rural areas by more generously investing in loan repayment programs.

Other financial factors, such as hiring bonuses or higher pay, are not a major factor in influencing individuals to practice in rural areas. This finding has important implications for rural health care systems as well as policy makers. Certainly, competitive compensation is necessary for the recruitment and retention of health care workers, and market forces cannot be ignored, but the results here indicate that financial incentives are not the biggest drivers of rural workforce recruitment.

Similarly—and again, somewhat counter to conventional wisdom—considerations about the area itself do not appear to be major drivers of health care professionals’ decision to practice in a rural setting once rural background is controlled. Undoubtedly, there is a high correlation between having grown up in a rural area and valuing the characteristics of rural areas, but once that correlation is controlled, as we have done in our model, the association between lifestyle and area considerations and rural practice is not substantial, suggesting that focusing on this may not be the most effective recruitment message. Likewise, after controlling for growing up in a rural area, family considerations are not a major driver of rural recruitment except among RNs.

Considerations about the way one can practice are important across these professions. Among 3 professions—physicians, APRNs, and RNs—being able to have autonomy in one’s practice is associated with working in a rural area. This may reflect the overall changing landscape of health care systems and consolidation that differ somewhat between rural and urban areas. Smaller health care facilities likely have more inherent autonomy for their workers; emphasizing (and preserving) this workplace characteristic may be effective during recruitment efforts, as well as in efforts aimed at retaining health care workers in rural areas. Finally, for physicians and APRNs, the desire to have a broad scope of practice drew people into rural work, suggesting that the more holistic, whole-person care more common in rural areas is a strong incentive for these health care professionals.

The rural health care landscape is changing rapidly, and it is not clear what the effect of these changes will be on the health care workforce. For example, the Centers for Medicare and Medicaid Services is implementing the Rural Emergency Hospital designation, which will incentivize rural hospitals to shut down inpatient services and only provide emergency, observation, and outpatient care.^17^ Given rural health care professionals’ desire to practice broadly, it is possible that such changes could present further challenges to recruitment and retention of rural health care professionals. It is also possible, however, that reducing demands and potential burnout could be a positive factor for rural health care workforce recruitment and retention.

### Limitations

This study has several limitations. First, our data rely on self-report of health care professionals and thus are potentially subject to recall bias. Further, respondents’ indication of where they grew up may be influenced more by their perception of that area’s characteristics than actual census information. Second, our data rely on reported (and corrected) addresses to identify practice location; these data may contain inaccuracies. Last, these data are from 1 state (Minnesota) and may not be generalizable to other states or geographic areas.

## Conclusions

The findings of this survey study suggest that there may be many factors that determine where health care professionals choose to live and work. Given the importance of rural health care worker shortages in contributing to rural health care inequities, understanding these factors, their relative importance, and their relative modifiability are important in influencing stakeholders’ strategic decision making. Such stakeholders include rural communities; local, state, and federal governmental policy makers; educational programs and institutions; and rural health care systems. Programs focusing on recruitment of rural residents into health care professions will likely yield long-term results, and more short-term modifiable factors include emphasizing practice characteristics, clinician autonomy, loan repayment programs, and scope of practice. Future research should continue to address the association of these factors as well as the relative effectiveness of interventions to modify them.

## Notes

Supplement 1. **eMethods.** Workforce Survey

Supplement 2.
Data Sharing Statement
