# Supplementary material for: Factors Associated With Health Care Professionals’ Choice to Practice in Rural Minnesota
Source: JAMA Netw Open. 2023 May 4;6(5):e2310332. doi: 10.1001/jamanetworkopen.2023.10332 (PMC10160870; doi:10.1001/jamanetworkopen.2023.10332)
Supplement: Supplement 1. — eMethods. Workforce Survey [file jamanetwopen-e2310332-s001.pdf]

Supplementary Online Content

Fritsma T, Henning-Smith C, Gauer JL, et al. Factors associated with health care professionals' choice to practice in rural Minnesota. *JAMA Netw Open*. 2023;6(5):e2310332. doi:10.1001/jamanetworkopen.2023.10332

eMethods. Workforce Survey

This supplementary material has been provided by the authors to give readers additional information about their work.

© 2023 Fritsma T et al. *JAMA Network Open*.

[Profession Name] Workforce Survey

The following is a health care workforce survey administered by the Minnesota Department of Health.

What is the purpose of the survey?

The Minnesota Department of Health (MDH) collects this data to inform state and national health care workforce policies, to understand emerging trends in health care, and to help ensure access to medical, oral, and behavioral health care services for all Minnesotans.

Do I have to take this survey?

Yes. You are legally required to provide this information. However, your responses **WILL NOT** affect your eligibility for a license in any way.

Are my survey responses protected?

Yes. The survey information you provide is classified as **private data on individuals,** which is protected under the Minnesota Government Data Practices Act, Minnesota Statutes, chapter 13. Your responses will be combined with others and reported by MDH in summary format only. We may only share your responses with authorized government entities or researchers, as allowed by law.

Who should I contact if I have questions?

Please contact the Minnesota Department of Health at (651) 201-3838 or toll free at (800) 366-5424.
Email: health.workforce@state.mn.us
Our office hours are 8 am to 4:30 pm M-F.

Click the NEXT button below to acknowledge that you have read the statements provided above to begin the survey.

< NEXT>

[Profession Name] Workforce Survey

Please note: It takes most people approximately 10 minutes to complete the survey. **If you take 30 minutes or longer, you may be logged out of the license renewal process. If this happens, your responses will be saved**, but you will need to close your browser and log back in at the board's website to continue. We appreciate your time in accurately responding to the survey.

What does the Minnesota Department of Health do with this data?

Pop-up: MDH uses the Health Workforce Survey data to study broad trends in the healthcare workforce. We combine your responses with others in order to understand the challenges facing your profession and the workforce more broadly. The data frequently informs policy discussions, program planning, and other MDH program requirements. Your responses are combined with others and are analyzed in the aggregate. The data you provide does not determine your eligibility for license renewal.

CORE QUESTIONS: Work Status, Education, Demographics

Work Status

1) Which of these best represents your **current** work status?

- ❍ Working in position that is related to my professional license (either within or outside Minnesota)
- ❍ Not working in a position that is related to my professional license

2) [If Q1 = Not working…] Which of these **best reflects** why you aren't working in a position related to your license?

- ❍ Retired
- ❍ Laid off or furloughed
- ❍ Between jobs and/or seeking work in a health care setting
- ❍ Working (or seeking work) outside health care
- ❍ Staying at home to provide care to family member(s) such as children, parents, or others
- ❍ Some other reason: ______________________________________

[If Q1 = "Not working…" skip to end of survey]

3) Approximately how many **hours** do you work in a typical week, including all duties of your job(s) (e.g., patient/client care time, documentation, administrative work, etc.)? (help)

_______ Hours per week (Enter a whole number. An estimate is fine.)

If your schedule varies widely from week to week, please provide an average.

4) In your position(s), do you provide any direct care to patients or clients?

- ❍ Yes
- ❍ No

5) About how many more years do you plan to work in this profession?

- ❍ Less than 1 year
- ❍ Between 1 and 5 years
- ❍ Between 5 and 10 years [Skip to Q7]
- ❍ More than 10 years [Skip to Q7]

6) [If Q5= “1 year or less” or Q5 = “Between 1 and 5 years”] What is the main reason you plan to leave the profession within the next five years? (help)

> Pop-up: If more than one of these reasons applies, select the one that best applies to you.

- ❍ To retire
- ❍ To leave the profession because of burnout or dissatisfaction
- ❍ To pursue a different career
- ❍ To pursue training in order to advance in my current or a related profession
- ❍ For family or other personal reasons
- ❍ For some other reason (please specify): ___________________________________

7) [Pharmacists, Physicians, Dentists, APRNs, LICSWs, LMFTs, LPCCs, Psychologists, Physical Therapists] Do you own (or co-own) your own business or an individual or group private practice?

- ❍ Yes
- ❍ No

8) In which setting do you typically work the most hours? (help)

> Pop-up: If your work site falls into more than one category, please choose the one that best applies. If you work at more than one site, choose the one where you work the most hours.

- ❍ **A remote location (e.g., home) to care for patients or clients via telemedicine, phone, and/or email**
- ❍ **Mobile clinic**
- ❍ **Academic** (Teaching / Research)
- ❍ **Clinic / Professional Office / Health Center / Ambulatory Care** (including behavioral health or substance abuse clinics, community health centers, FQHCs, surgery centers, dental offices, private practice, health and wellness facilities, urgent care, or walk-in, retail, or convenience clinics)
- ❍ **Community / Faith-Based Organization** (including community collaboratives, non-profit organizations, or social service agencies)
- ❍ **Correctional Facility**
- ❍ **Home Health Care** (including any medical or behavioral health care that is provided in patients’ or clients’ homes including Home Infusion)
- ❍ **Hospital—Inpatient or Outpatient** (including day surgery, emergency department, behavioral health/psychiatric, specialty, transitional/rehabilitation unit)
- ❍ **Long-Term Care Facility** (including assisted living, hospice, rehabilitation, group homes, residential care, skilled nursing, or transitional/sub-acute care)
- ❍ **Pharmacy** (including hospitals/clinics/nursing facilities, independent community pharmacies, mail service pharmacy, or chain pharmacies)
- ❍ **Public Health Agency** (including city/county health board, or city/county/state public health entity)
- ❍ **School (Pre-K though 12)**
- ❍ **Other (please specify): ____**

10) [For Pharmacists and Pharmacy Technicians only; and if Q9 = "Pharmacy"] Please select pharmacy type. (help)

> Pop-up: If your work site falls into more than one category, please choose the category you think best applies.

- ❍ Chain community pharmacy
- ❍ Centralized service pharmacy
- ❍ Clinic-based pharmacy
- ❍ Compounding pharmacy
- ❍ Hospital-based pharmacy
- ❍ Independent community pharmacy
- ❍ Mail service pharmacy
- ❍ Medication therapy management
- ❍ Nuclear pharmacy
- ❍ Supermarket or mass merchandiser pharmacy
- ❍ Telepharmacy

11) [For Dentists, Dental Hygienists, Dental Assistants; and if Q9 = "Clinic/Professional Office/Health Center/Ambulatory Care"] Please select clinic/office/health center type. (help)

> Pop-up: If your work site falls into more than one category, please choose the category you think best applies.

- ❍ Solo private practice
- ❍ Small group private practice (2-4 dentists)
- ❍ Large group private practice (5+ dentists)
- ❍ Community based non-profit (e.g. church, homeless shelter, early childhood education, etc.)
- ❍ Community Health Center (CHC)/Federally Qualified Health Center (FQHC)
- ❍ Mobile dental clinic (e.g. Southside, Operation Grace)
- ❍ Dental education institution clinic

Education

12) [Not physicians or dentists] What is the highest degree you have completed?

- ❍ High school diploma
- ❍ Certificate (such as technical or vocational)
- ❍ Professional diploma (such as in nursing)
- ❍ Some college, no degree
- ❍ Associate degree
- ❍ Certificate, certification or other credential (post Associate degree)
- ❍ Bachelor's degree
- ❍ Certificate, certification or other credential (post Bachelor's degree)
- ❍ Master's degree
- ❍ Certificate, certification or other credential (post Master's degree)
- ❍ Doctorate or professional degree

13) [Not physicians or dentists] Where did you complete this degree?

Dropdown box with each state, plus Canada, plus "Another country" at the bottom.

14) [LPNs, RNs, APRNs, LSW, LGSW, LISW, LICSW, LPCS, LPCCS, LADCs, LMFTS, PTs, Pharmacists, LPs]
Was this degree in nursing / a mental health related field / pharmacy / physical therapy?

- ❍ Yes
- ❍ No

15) [LPNs, RNs, APRNs, LSW, LGSW, LISW, LICSW, LPCS, LPCCS, LADCs, LMFTS, PTs, Pharmacists, LPs]
What was the *lowest* degree you completed that qualified you to work as a[n] nurse / social worker / licensed counselor / marriage and family therapist / pharmacist / physical therapist / psychologist?

- ❍ Same as my highest degree
- ❍ High school diploma
- ❍ Certificate (such as technical or vocational)
- ❍ Professional diploma (such as in nursing
- ❍ Some college, no degree
- ❍ Associate degree
- ❍ Certificate, certification or other credential (post Associate degree)
- ❍ Bachelor's degree
- ❍ Certificate, certification or other credential (post Bachelor's degree)
- ❍ Master's degree
- ❍ Certificate, certification or other credential (post Master's degree)
- ❍ Doctorate or professional degree
- ❍ Certificate, certification or other credential (post Master's degree)
- ❍ Doctorate or professional degree

16) [If Q15 NE "Same as my highest degree"] Was this degree in nursing / a mental health related field / pharmacy / physical therapy?

- ❍ Yes
- ❍ No

Dental Hygienist and Dental Therapist Extra Questions (I've kept these questions un-numbered)

Remove extra questions for dentists.
Remove extra questions for dental assistants.

[Dental Hygienists only] Do you have a written collaborative agreement with a dentist allowing you to provide services outside a traditional dental office or in a community/non-traditional setting (MN Statute 150A.10, Subd. 1a)?

- ☐ Yes
- ☐ No
- ☐ Don't know

[If Yes] How often do you use your collaborative agreement in your current job?

- ☐ Never
- ☐ Up to 25 percent of my time
- ☐ Between 25 and 50 percent of my time
- ☐ Between 50 and 75 percent of my time
- ☐ More than 75 percent of my time

[Dental Therapists only] In general, what percentage of time do you typically perform dental hygiene procedures?

- ☐ None—I spend all of my time doing dental therapy procedures
- ☐ Up to 25 percent of my time
- ☐ Between 25 and 50 percent of my time
- ☐ Between 50 and 75 percent of my time
- ☐ More than 75 percent of my time

[Dental Therapists only] Are you certified as an advanced dental therapist (ADT)?

- ☐ Yes
- ☐ No
- ☐ In process
- ☐ Not interested

[If Yes In general, what percent of your time do you typically perform procedures, including evaluations and extractions authorized strictly for Advanced Dental Therapists (procedures relating to CDT codes D0120, D0140, D0145, and D7140)?

- ☐ None
- ☐ Up to 25 percent of my time
- ☐ Between 25 and 50 percent of my time
- ☐ Between 50 and 75 percent of my time
- ☐ More than 75 percent of my time

[If Yes] In general, what percent of your time do you work under general supervision when the collaborative dentist is not physically present at your practice location (including clinic and community settings)?

- ☐ None
- ☐ Up to 25 percent of my time
- ☐ Between 25 and 50 percent of my time
- ☐ Between 50 and 75 percent of my time
- ☐ More than 75 percent of my time

17) Which racial/ethnic categories apply to you? (*Check all that apply*.)

- ☐ African
- ☐ Black/African American
- ☐ American Indian or Alaskan Native
- ☐ Asian—South Asian
- ☐ Asian—Southeast Asian
- ☐ Asian—Other (please specify other Asian race): ___________________
- ☐ Hispanic/Latino
- ☐ Middle Eastern/North African (MENA)
- ☐ White
- ☐ Other (please specify your other race): _________________________________

18) [If Q5 is not "No"] Other than English, what languages **do you personally speak** in your practice? (*Check all that apply. Do not include languages spoken only through a medical interpreter.*)

- ☐ None—English only
- ☐ Arabic
- ☐ Chinese (Mandarin/Cantonese)
- ☐ Hmong
- ☐ Karen
- ☐ Khmer
- ☐ Lao
- ☐ Oromo
- ☐ Russian
- ☐ American Sign Language
- ☐ Somali
- ☐ Spanish
- ☐ Swahili
- ☐ Vietnamese
- ☐ Other (please specify): _________________________________

SPECIAL MODULE (This whole module should be asked of people for whom Q4 NE “No” [anyone who is providing patient care])

---

19) Which of the following **best prepared you** to work with people from a variety of backgrounds when providing care (sometimes referred to as “culturally competent” care)?

- ❍ None
- ❍ Informal learning on the job
- ❍ Formal on-the-job training (e.g., seminars, preceptorship or mentorship)
- ❍ Course work or training in my formal educational program
- ❍ Continuing education or professional development coursework
- ❍ Does not apply—culturally competent care is not part of my job

20) Why did you select that response?

____

21) Approximately how much of the care that **you personally** provide is via telemedicine?

- ❍ None
- ❍ Up to 10 percent
- ❍ Between 10 and 25 percent
- ❍ Between 25 and 50 percent
- ❍ Between 50 and 75 percent
- ❍ Between 75 percent and 100 percent
- ❍ All of the care I provide is via telemedicine

22) [If Q21 NE “None”] Not including those who are on vacation or at a cabin, how often do you provide care via telemedicine to people who live in areas that are more rural than where you practice?

- ❍ At least daily or weekly
- ❍ At least monthly
- ❍ Occasionally, but less than monthly
- ❍ Never
- ❍ Don’t know

23) Which of these best describes the type of area where you grew up? help

Pop-up: If you grew up in more than one type of area, choose the one you most identify with.

- ❍ Large metropolitan or surrounding
- ❍ Small city
- ❍ Small town or rural area

24) Which of these best describes the type of area where you live now?

- ❍ Large metropolitan or surrounding
- ❍ Small city
- ❍ Small town or rural area

25) If [Q23 = "Large metropolitan or surrounding" AND Q24 = "Large metropolitan or surrounding"] Did you ever *seriously* consider living in a small town or rural area?

- ❍ Yes, I seriously considered it
- ❍ I have lived in a small town or rural area
- ❍ No

26) If [Q23 = "Small town or rural area" and Q24 = "Small town or rural area"] Did you ever *seriously* consider living in a large metropolitan area?

- ❍ Yes, I seriously considered it
- ❍ I have lived in a large metropolitan area
- ❍ No

27) Think back to how you made the decision to live in your general area. How important were each of the following considerations?

	**Very important**	**Somewhat important**	**Not important at all**	**Did not apply to me**
The lifestyle in this area				
Whether my partner/spouse would have job opportunities				
Whether I would have autonomy in my work				
Whether this would be a good place to raise children				
An internship, clinical training, or residency exposed me to what it's like to work in this area, or a similar one				
Living close to family or friends				
Whether I would be able to work with certain types of patients or clients				
A financial incentive to live here, such as higher pay or a hiring bonus				
The quality of life in this area				
Whether I could specialize in certain types of care in this area				
Whether I could (or did) receive a loan forgiveness award				
My educational program emphasized caring for patients/clients in an area like this (e.g., urban or rural)				
Whether or not I could find a romantic partner in this area				
Whether I could have a broad scope of practice (variety) in my work				
Whether the community here would be a good fit for me				
A "calling" to work with patients/clients in this type of area				
Other (specify):__________________				

28) When thinking about the area in which you live (major metro area; small city; small town/rural), which of these would you say influenced your decision the **most**?

- ❍ Family considerations
- ❍ The way I could practice/work in this area
- ❍ Education/training experiences
- ❍ Things about the area itself
- ❍ Financial incentive(s)
- ❍ Something else: ____

29) [Optional] Please describe in your own words how you chose to live in the area where you're currently living.
____

APPENDIX

List of professions surveyed

**Profession Name**	**Abbreviation**
Counselors—Licensed Alcohol and Drug Counselors	LADC
Counselors—Licensed Professional Clinical Counselors	LPCC
Counselors—Licensed Professional Counselors	LPC
Dental Assistants	A
Dental Hygienists	H
Dental Therapists	T
Dentists	D
Licensed Marriage and Family Therapists	LMFT
Nurses—Advanced Practice Registered Nurses	APRN
Nurses—Licensed Practical Nurses	L
Nurses—Registered Nurses	R
Pharmacists	PHARM
Pharmacy Technicians	PHARMT
Physical Therapist Assistants	PTA
Physical Therapists	PT
Physician Assistants	PA
Physicians	PY
Psychologists	LP
Respiratory Therapists	RT
Social Workers—Licensed Graduate Social Workers	LGSW
Social Workers—Licensed Independent Clinical Social Workers	LICSW
Social Workers—Licensed Independent Social Workers	LISW
Social Workers—Licensed Social Workers	LSW
