# Supplementary material for: Factors Associated With Health Care Professionals’ Choice to Practice in Rural Minnesota
Source: JAMA Netw Open. 2023 May 4;6(5):e2310332. doi: 10.1001/jamanetworkopen.2023.10332 (PMC10160870; doi:10.1001/jamanetworkopen.2023.10332)
Supplement: Supplement 2. — Data Sharing Statement [file jamanetwopen-e2310332-s002.pdf]

Data Sharing Statement

Fritsma. Factors Associated With Health Care Professionals' Choice to Practice in Rural Minnesota. *JAMA Netw Open*. Published May 04, 2023.
doi:10.1001/jamanetworkopen.2023.10332

Data
Data available: No
